# Neurological intervention transition model for dynamic prediction of good outcome in spontaneous subarachnoid haemorrhage

**DOI:** 10.1038/s41598-024-51684-6

**Published:** 2024-01-18

**Authors:** Yiming Luo, Stephen John Payne

**Affiliations:** 1 Institute of Biomedical Engineering, Department of Engineering Science, University of Oxford, Oxford, OX3 7DQ UK; 2 Institute of Applied Mechanics, National Taiwan University, Taipei, 106 Taiwan

**Keywords:** Prognosis, Outcomes research, Biomedical engineering, Risk factors, Stroke

## Abstract

Deterioration of neurovascular conditions can be rapid in patients with spontaneous subarachnoid haemorrhage (SAH) and often lead to poor clinical outcomes. Therefore, it is crucial to promptly assess and continually track the progression of the disease. This study incorporated baseline clinical conditions, repeatedly measured neurological grades and haematological biomarkers for dynamic outcome prediction in patients with spontaneous SAH. Neurological intervention, mainly aneurysm clipping and endovascular embolisation, was also incorporated as an intermediate event in developing a neurological intervention transition (NIT) joint model. A retrospective cohort study was performed on 701 patients in spontaneous SAH with a study period of 14 days from the MIMIC-IV dataset. A dynamic prognostic model predicting outcome of patients was developed based on combination of Cox model and piecewise linear mixed-effect models to incorporate different types of prognostic information. Clinical baseline covariates, including cerebral oedema, cerebral infarction, respiratory failure, hydrocephalus and vasospasm, as well as repeated measured Glasgow Coma Scale (GCS), glucose and white blood cell (WBC) levels were covariates contributing to the optimal model. Incorporation of neurological intervention as an intermediate event increases the prediction performance compared with baseline joint modelling approach. The average AUC of the optimal model proposed in this study is 0.7783 across different starting points of prediction and prediction intervals. The model proposed in this study can provide dynamic prognosis for spontaneous SAH patients and significant potential benefits in critical care management.

## Introduction

Spontaneous SAH is characterised by sudden bleeding into the subarachnoid space due to non-traumatic causes, mostly arising from a ruptured cerebral aneurysm. It is considered as a medical emergency due to various associated complications and its life-threatening nature, such that the 28-day mortality rate is reported as high as 41.7%^1^. Close monitoring of disease progression and prediction of adverse clinical outcomes in the critical care are thus required to identify risks of deterioration and track changes in neurological status, which can evolve rapidly. This allows for timely neurological intervention, adjustments in treatment strategies, and proactive preventive measures to optimise critical care management and improve patient outcomes.

The prognostication of spontaneous SAH is intricate and multi-factorial. In clinical practice, the prognosis is commonly made through patients’ medical conditions and various assessments. These medical conditions include underlying conditions, e.g., hypertension, and diabetes, as well as complications, e.g., re-bleeding, vasospasm, hydrocephalus, and cerebral oedema. Clinical assessments used for prognostication of spontaneous SAH include clinical examinations, e.g., GCS to assess the level of consciousness, radiological investigations on brain imaging, haematological biomarkers, e.g., white blood cell (WBC) count, and vital signs, e.g., blood pressure and oxygen saturation, that provide information about a patient's physiological status.

Clinical tools for prognosis of spontaneous SAH have been mostly developed based on baseline medical conditions and clinical assessments. The Hunt and Hess scale and the World Federation of Neurological Surgeons (WFNS) scale are the two most widely used prognostic scoring systems for SAH, focusing on the patient’s level of consciousness and neurological deficits^2,3^. Another scoring system, the Fisher grade or modified Fisher grade uses findings of the amount of blood and intraventricular haemorrhage (IVH) on brain computed tomography (CT) scans to assess the severity of SAH^4,5^. A multidimensional tool, the FRESH score was also proposed for prognostication of outcomes after spontaneous SAH, incorporating Hunt and Hess and APACHE-II physiologic scores on admission, age and rebleeding within 48 h^6^.

The prognostication of spontaneous SAH can be potentially improved in several aspects. Firstly, these prognostic scoring systems focus on a specific aspect of medical conditions, particularly patient’s consciousness level and neurological deficits. Although this could be the most critical aspect of prognosis and is valuable in clinical practice for risk identification, other prognostic factors can provide additional prognostic information and should be included in multi-factorial prognostic models for more comprehensive and customised prognostication.

Secondly, vital signs and neurological status, usually measured by GCS score, are repeatedly measured and recorded routinely to monitor disease progression and assess treatment response. They provide up-to-date dynamic prognostic information on patients’ evolving conditions. Incorporation of up-to-date dynamic prognostic information in prognostic modelling enables clinicians to determine and adjust treatment plans effectively and provide realistic expectations on patients’ clinical outcomes.

Finally, intermediate events, e.g., neurological interventions and occurrences of complications, can significantly impact the progression and clinical outcomes. Incorporating these intermediate events in prognostication of spontaneous SAH can help improve the prediction accuracy of prognostic models, allowing for a more comprehensive and dynamic approach to prognostication to enhance the management and prognosis of patients suffering spontaneous SAH.

This study aims to develop a dynamic prognostic model for spontaneous SAH to improve prognostication as well as explore the prognostic values of neurological interventions when jointly analysed with baseline and dynamic covariates. For the multi-factorial nature of prognostication, various clinical conditions on admission, e.g., demographics and underlying medical conditions, will be incorporated as baseline covariates, which are unaltered during the study period, for prognostic modelling.

Dynamic prognostic information, provided by repeated measured vital signs, haematological biomarkers and neurological assessments, as well as a neurological intervention, is then simultaneously analysed and modelled with baseline covariates for monitoring the disease progression of each patient. The proposed prognostic model takes various clinical conditions on admission, multiple dynamic factors providing up-to-date prognostic information, and the effect of neurological interventions into account for prognostication. These advantages make it an novel customised clinical tool for risk assessment, customised treatment optimisation, and disease progression monitoring.

## Methods

### Data collection and visualisation

Patient recruited in this study were selected from MIMIC-IV, a publicly available database sourced from the electronic health record (EHR) of the Beth Israel Deaconess Medical Center between 2008 and 2019^7^. 766 patients with spontaneous SAH as the primary diagnosis were initially included in the study, identified by ICD-9-CM code 430 or ICD-10-CM code I60 and their specifiers. 65 patients were excluded due to too short hospital stay (< 24 h) or having no identified records in vital signs and neurological assessments.

A final total of 701 patients were included in this study, all patients aged 18 and over. Among them, 409 (58.35%) patients were females, and the median age was 59 (IQR: 50–70). Essential hypertension is the most common clinical condition (340, 48.50%), followed by hydrocephalus (221, 31.53%), respiratory failure (149, 21.56%), and cerebral oedema (131, 18.69%). Clinical outcomes were measured by discharge destinations at the end of study period. Good outcome (343, 48.93%) was defined by returning home or rehabilitation, while poor outcome (358, 51.07%) includes mortality, long-term acute care, and hospice care. The clinical characteristics of the dataset are shown in Table 1. Demographics, e.g., age and female, are recorded in patients’ clinical information in the database, while underlying conditions and complications are identified with ICD-9-CM and ICD-10-CM codes.

**Table 1:** Clinical characteristics of included dataset.

${\text{Covariate}}$		$\mathrm{All }\,({\text{n}}=701)$	$\mathrm{Good\,outcome }\,({\text{n}}=343)$	$\mathrm{Poor\, outcome }\,({\text{n}}=358)$
${\text{Demographics}}$	${\text{Age}}$	Median: 59 (IQR: 50—70)	Median: 49.25 (IQR: 56—68)	Median: 61 (IQR: 51—73)
	${\text{Female}}$	409 (58.35%)	192 (55.98%)	217 (60.61%)
$\mathrm{Underlying \,conditions}$	${\text{Alcohol}}$	23 (3.28%)	9 (2.62%)	14 (60.61%)
	${\text{Tobacco}}$	132 (18.83%)	68 (19.83%)	64 (3.91%)
	${\text{Diabetes}}$	76 (10.84%)	32 (9.33%)	44 (12.29%)
	${\text{Essential}}$ ${\text{hypertension}}$	340 (48.50%)	154 (44.90%)	186 (51.96%)
${\text{Complications}}$	$\mathrm{Cerebral}\; \mathrm{oedema}$	131 (18.69%)	24 (7.00%)	107 (29.89%)
	$\mathrm{Cerebral} \; \mathrm{infarction}$	69 (9.84%)	15 (4.37%)	54 (15.08%)
	$\mathrm{Respiratory\, failure}$	149 (21.56%)	11 (3.21%)	138 (38.55%)
	${\text{Hydrocephalus}}$	221 (31.53%)	34 (3.21%)	187 (52.23%)
	${\text{Vasospasm}}$	55 (7.85%)	15 (4.37%)	40 (11.17%)

Six indices were included as dynamic covariates to be selected for prognostic modelling, including one neurological grade, i.e., GCS score, three haematological biomarkers, i.e., creatinine, WBC, glucose, and two vital signs, i.e., systolic blood pressure (SBP) and oxygen saturation (SpO2), which had been included and shown to be valuable in SAH prognosis research^8–13^. Table 2 presents the average values and standard deviations of these dynamic covariates, calculated among all observations of patients for both good and poor outcome groups to provide a general statistical overview of each dynamic covariate. As the frequency of each index measure can vary depending on severity of patients’ clinical conditions, different phases of the disease and the judgement of healthcare providers, we have incorporated daily average values of dynamic covariates into prognostic modelling to normalise the frequency of measurements across patients, thereby reducing potential biases due to variations in measurement frequencies.

**Table 2:** Statistics of all observations of six dynamic covariates.

${\text{Covariate}}$		${\text{All}}$	$\mathrm{Good\, outcome}$	$\mathrm{Poor\, outcome}$
$\mathrm{Neurological\, Score}$	$\mathrm{GCS\, Score}$	$11.89\pm 3.72$	$14.57\pm 3.72$	$10.58\pm 3.84$
$\mathrm{Haematological\, Biomarkers}$	${\text{Creatinine}}$ (mg/dL)	$0.75\pm 0.72$	$0.84\pm 1.06$	$0.70\pm 0.37$
	$\mathrm{WBC }\,(10^9/{\text{L}})$	$11.57\pm 5.19$	$9.68\pm 4.04$	$12.68\pm 5.46$
	$\mathrm{Glucose }\,({\text{mg}}/{\text{dL}})$	$129.29\pm 37.74$	$117.69\pm 32.49$	$136.30\pm 38.95$
$\mathrm{Vital\, Signs}$	$\mathrm{SBP }\,({\text{mmHg}})$	$141.99\pm 20.76$	$134.66\pm 17.03$	$144.03\pm 21.24$
	${\text{SpO}}2\, (\mathrm{\%})$	$97.08\pm 3.54$	$96.72\pm 4.67$	$97.26\pm 2.82$

**Figure 1:**
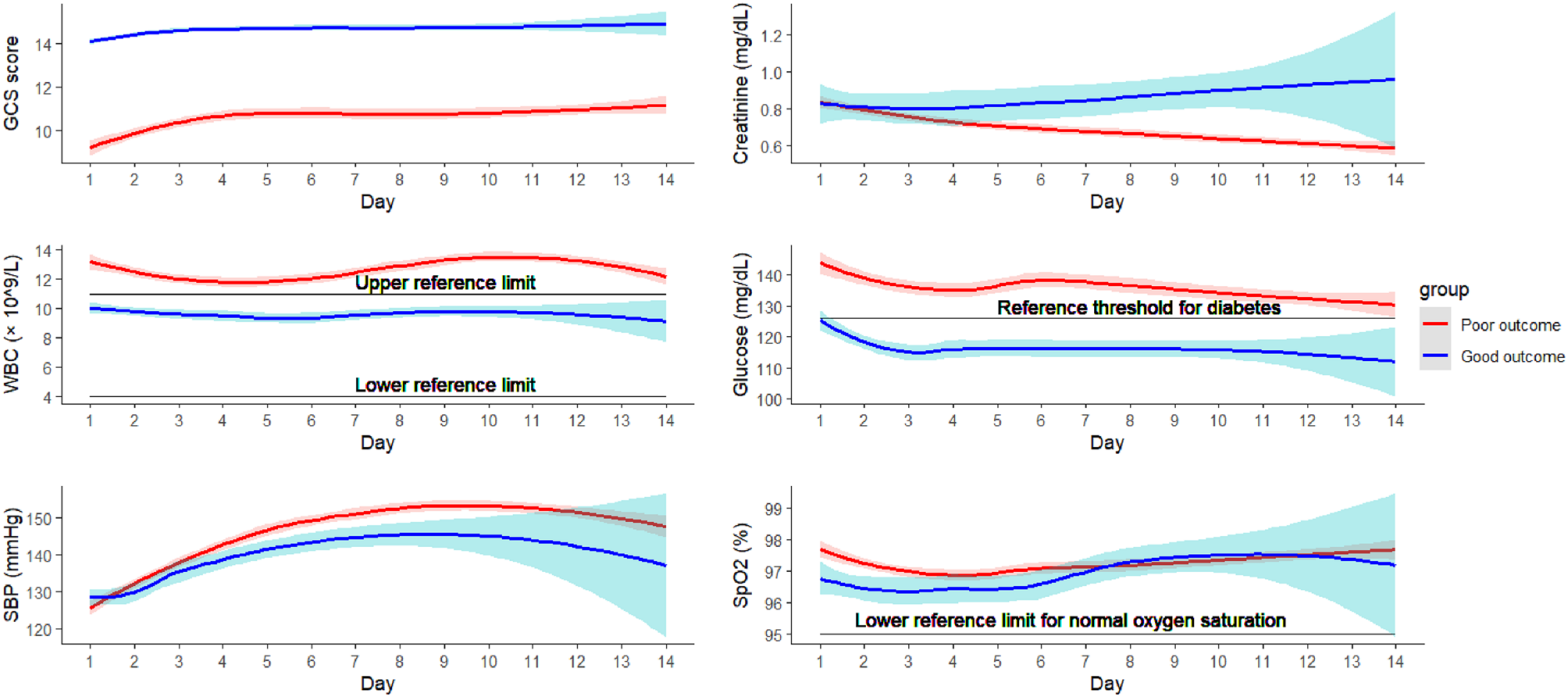
Trajectories of six dynamic prognostic covariates with LOESS method.

Figure 1 visualises the trajectories and corresponding 95% confidence interval of these dynamic covariates during the study period with locally estimated scatter-plot smoothing (LOESS) method. The LOESS method was separately applied to each group, estimating a smoothed curve that best represents the trend of each dynamic covariate for each group.

According to the trajectory visualisation in Fig. 1 and statistics shown in Table 2, GCS score is a discriminatively powerful dynamic covariate between two groups, while the trajectories of WBC and glucose also exhibit good discrimination between good outcome and poor outcome. The other three covariates, however, do not exhibit discriminative power. Visualisation of the trajectories of dynamic prognostic covariates allow to observe their patterns and trends for each group and provide an initial insight into the dynamic nature of these covariates. The significance of prognostic values of these dynamic covariates needs to be jointly analysed with baseline covariates and intermediate events, which will be presented later.

### Prognostic modelling

The prognostic modelling process starts with examining the effects of baseline covariates on clinical outcomes, which can be modelled using a Cox proportional hazard model. The Cox model is the most widely used method in survival analysis and can be used to investigate the effects of baseline covariates on clinical outcomes, given by^14^:

1 $$h_{i} (t) = h{}_{0}(t)\exp \left( {\sum\limits_{j} {\gamma_{j} } \omega_{{j_{i} }} } \right)$$

where ${h}_{i}(t)$ denotes the instantaneous rate of experiencing a good clinical outcome for patient *i* at time *t,* while ${\omega }_{{j}_{i}}$ is the value of the ${j}^{th}$ baseline covariate for patient i with corresponding coefficient ${\gamma }_{j}$. A positive coefficient indicates increasing chance of a good outcome, whereas a negative coefficient implies higher risk of poor outcomes. The effect of ${j}^{th}$ baseline covariate on outcome is measured by hazard ratio (HR), computed as ${\text{exp}}({\gamma }_{j})$.

Estimated HRs of all analysed baseline covariates are less than 1, indicating lower changes of good outcomes, as shown in Table 3. Results in univariate analysis revealed effects of single covariates on clinical outcome, while multivariate analysis considered the simultaneous effects of multiple covariates, estimating the effect of each covariate while accounting for other covariates. Among these baseline covariates, cerebral oedema, cerebral infarction, respiratory failure, hydrocephalus and vasospasm are significant in univariate survival analysis and remain significant in multivariate survival analysis. It is noted that HRs of significant baseline covariates increase towards 1 in multivariate analysis compared to univariate analysis, indicating that their effects on clinical outcomes are less pronounced when other variables are accounted for. With multiple baseline covariates obtained, the prognostic model doesn’t need to rely on a single baseline covariate to predict outcomes that the effect of that covariate may be overestimated. Instead, we can have a more comprehensive understanding of the patient’s condition emerges to make more comprehensive and accurate predictions on prognosis.

**Table 3:** Results of univariate and multivariate survival analysis of baseline covariates.

${\text{Covariate}}$	$\mathrm{Univariate\, analysis}$			$\mathrm{Multivariate\, analysis}$		
	${\text{HR}}$	$95\mathrm{\%\,CI}$	$\mathrm{P\, value}$	${\text{HR}}$	$95\mathrm{\%\,CI}$	$\mathrm{P\, value}$
${\text{Age}}$	$0.9944$	$0.9870-1.0019$	$0.144$	$-$	$-$	$-$
${\text{Female}}$	$0.8856$	$0.7155-1.0961$	$0.264$	$-$	$-$	$-$
${\text{Alcohol}}$	$0.6952$	$0.3586-1.3480$	$0.282$	$-$	$-$	$-$
${\text{Tobacco}}$	$0.9890$	$0.7584-1.2898$	$0.935$	$-$	$-$	$-$
${\text{Diabetes}}$	$0.7400$	$0.5142-1.0649$	$0.105$	$-$	$-$	$-$
$\mathrm{Essential \,hypertension}$	$0.8566$	$0.6924-1.0598$	$0.154$	$-$	$-$	$-$
$\mathrm{Cerebral} \; \mathrm{oedema}$	$\mathbf{0.2661}$	$\mathbf{0.1757-0.4031}$	$\mathbf{<0.001}$	$\mathbf{0.5626}$	$\mathbf{0.3680-0.8600}$	$\mathbf{0.008}$
$\mathrm{Cerebral}\; \mathrm{infarction}$	$\mathbf{0.2913}$	$\mathbf{0.1736-0.4889}$	$\mathbf{<0.001}$	$\mathbf{0.3644}$	$\mathbf{0.2169-0.6124}$	$\mathbf{<0.001}$
$\mathrm{Respiratory\, failure}$	$\mathbf{0.0767}$	$\mathbf{0.0420-0.1400}$	$\mathbf{<0.001}$	$\mathbf{0.1345}$	$\mathbf{0.0727-0.2488}$	$\mathbf{<0.001}$
${\text{Hydrocephalus}}$	$\mathbf{0.1280}$	$\mathbf{0.0897-0.1828}$	$\mathbf{<0.001}$	$\mathbf{0.1821}$	$\mathbf{0.1271-0.2609}$	$\mathbf{<0.001}$
${\text{Vasospasm}}$	$\mathbf{0.3316}$	$\mathbf{0.1975-0.5566}$	$\mathbf{<0.001}$	$\mathbf{0.4092}$	$\mathbf{0.2430-0.6891}$	$\mathbf{<0.001}$

Values presented in bold indicate statistical significance.

As dynamic prognostic covariates can be measured with errors and influenced by caregivers, e.g., human biases on neurological assessments, linear mixed-effect models (LMM) are adopted to model the longitudinal properties of these dynamic covariates to handle unobserved heterogeneity. Moreover, since each patient corresponds to repeated measurements of dynamic covariates, the measurements within the same patient are likely to be correlated. An LMM can account for this within-subject correlation by including random effects. Fixed-effect and random-effect terms in an LMM for a dynamic covariate respectively describe its population-level mean trajectory and individual-specific deviations.

The longitudinal pattern of the ${j}^{th}$ dynamic covariate for the ${i}^{th}$ patient at time *t* is thus given by:

2 $$y_{{j_{i} }} (t) = m_{{j_{i} }} (t) + \varepsilon_{{j_{i} }} (t) = \beta_{j} x_{{j_{i} }}^{T} (t) + b_{{j_{i} }} z_{{j_{i} }}^{T} (t) + \varepsilon_{{j_{i} }} (t)$$

where ${y}_{{j}_{i}}(t)$ denotes the observed value of ${j}^{th}$ dynamic covariate, measured with error, while ${m}_{{j}_{i}}(t)$ is the corresponding unobserved true value, composed of fixed-effect term ${\beta }_{j}{x}_{{j}_{i}}^{T}(t)$ representing the overall trend for all patients, and random-effect term ${b}_{{j}_{i}}{z}_{{j}_{i}}^{T}(t)$ for explaining patient-specific deviations from the overall trend.

These dynamic covariates are then simultaneously modelled with baseline covariates to incorporate up-to-date dynamic prognostic information into prognostication with joint modelling approach^15^. A survival sub-model incorporating multiple dynamic and baseline covariates is given by:

3 $$h_{i} (t) = h{}_{0}(t)\exp (\gamma^{T} \omega_{i} + \sum\limits_{j} {\alpha_{j} } m_{{j_{i} }} (t))$$

In comparison with Eqs. (1, 3) simultaneously analyses the effects of baseline and dynamic covariates on clinical outcomes by incorporating longitudinal sub-models for these dynamic covariates as described in Eq. (2).

Next, there is a further source of prognostic information that can be incorporated in prognostic modelling, which are intermediate events, specifically neurological interventions in this study. For the prognostication of spontaneous SAH, neurological interventions can have a direct impact on disease progression and clinical outcomes, and can be incorporated into modelling framework for more comprehensive prognostication.

To account for the effect of neurological interventions on disease progression, the longitudinal sub-model of prognostic modelling, denoted by Eq. (2), can be reformulated as:

4 $$y_{{j_{i} }} (t) = m_{{j_{i} }} (t) + \varepsilon_{{j_{i} }} (t) = \left\{ \begin{gathered} \beta_{j} x_{{j_{i} }}^{T} (t) + b_{{j_{i} }} z_{{j_{i} }}^{T} (t) + \varepsilon_{{j_{i} }} (t){,} \hfill \\ \beta_{j} x_{{j_{i} }}^{T} (t) + b_{{j_{i} }} z_{{j_{i} }}^{T} (t) + \tilde{\beta }_{j} \tilde{x}_{{j_{i} }}^{T} (t_{ + } ) + \tilde{b}_{{j_{i} }} \tilde{z}_{{j_{i} }}^{T} (t_{ + } ) + \varepsilon_{{j_{i} }} (t){,} \hfill \\ \end{gathered} \right.\begin{array}{*{20}c} {0 < t < \tilde{t}_{i} } \\ {t \ge \tilde{t}_{i} } \\ \end{array}$$

where $\tilde{t}_{i}$ is the time of the neurological intervention of the ${i}^{th}$ patient, while $t_{ + }$ denotes the time relative to neurological intervention. For the ${i}^{th}$ patient, ${t}_{+}=max(0,t-\widetilde{{t}_{i}})$. The effects of neurological interventions are modelled as additive terms on both overall population-level trend and individual-specific terms of dynamic covariates after the time point of a neurological intervention.

Corresponding, Eq. (3) can be extended as:

5 $$h_{i} (t) = \left\{ \begin{gathered} h{}_{0}(t)\exp (\gamma^{T} \omega_{i} + \sum\nolimits_{j} {\alpha_{j} {(}\beta_{j} x_{{j_{i} }}^{T} {(}t{)} + b_{{j_{i} }} z_{{j_{i} }}^{T} {(}t{))})} , \hfill \\ h{}_{0}(t)\exp (\gamma^{T} \omega_{i} + \sum\nolimits_{j} {\alpha_{j} {(}\beta_{j} x_{{j_{i} }}^{T} {(}t{)} + b_{{j_{i} }} z_{{j_{i} }}^{T} {(}t{)} + \tilde{\beta }_{j} \tilde{x}_{{j_{i} }}^{T} {(}t_{ + } {)} + \tilde{b}_{{j_{i} }} \tilde{z}_{{j_{i} }}^{T} {(}t_{ + } {))})} , \hfill \\ \end{gathered} \right.\begin{array}{*{20}c} {0 < t < \tilde{t}_{i} } \\ {t \ge \tilde{t}_{i} } \\ \end{array}$$

Equation 5 describes the prognosis of spontaneous SAH into two states, obtained from the combination of Eqs. (3 and 4). Patients having not received neurological interventions are regarded in the first state, while patients already treated with neurological interventions are in the second state. $\tilde{t}_{i}$ is the time point of state transition. Thus, this model is termed as neurological intervention transition (NIT) joint model, and its performance as a prognostic model will be compared with baseline joint models that do not include neurological interventions in prognostic modelling.

Parameters included in this model are estimated using the Bayesian approach, wherein the inference relies on a joint posterior distribution as the product of the observed data’s joint likelihood and prior distribution. In this study, prior beliefs on the parameters in joint modelling framework are from the values of parameters separately estimated in survival and longitudinal sub-models. The Bayesian approach is implemented via Markov chain Monte Carlo (MCMC) methods, with Gibbs and Metropolis–Hastings algorithms for sampling from distributions.

## Results

### Simultaneous analysis on longitudinal and survival data

The prognostic value of each dynamic covariate was measured by its HR, calculated by the exponential of ${\alpha }_{j}$, when jointly analysed and adjusted for the five significant baseline covariates in Table 3. Table 4 presents the HRs of included dynamic covariates with both baseline and proposed NIT joint modelling approach.

**Table 4:** Results of dynamic covariates in baseline and NIT joint modelling approach.

${\text{Covariate}}$		$\mathrm{Baseline} \; \mathrm{joint \, model}$			$\mathrm{NIT \, joint \, model}$		
		${\text{HR}}$	$95\mathrm{\%}\;{\rm CI}$	$P\;\mathrm{ value}$	${\text{HR}}$	$95\mathrm{\%}\;{\rm CI}$	$P\;\mathrm{ value}$
${\text{Univariate}}$	$\mathrm{GCS \, score}$	$2.7379$	$2.0044-3.7398$	$<0.001$	$3.2802$	$2.2559-4.7696$	$<0.001$
	$\mathrm{Creatinine \, abnormality}$	$1.1085$	$0.7548-1.6280$	$0.584$	$1.1087$	$0.7780-1.5799$	$0.580$
	$\mathrm{WBC \, abnormality}$	$0.7847$	$0.5515-1.1167$	$0.170$	$0.7954$	$0.5695-1.1110$	$0.197$
	$\mathrm{Hyperglycemia }\;({\text{Glucose}}\ge 126\;{\text{mg}}/{\text{dL}})$	$0.7474$	$0.4967-1.1247$	$0.151$	$0.7703$	$0.5031-1.1793$	$0.213$
	${\text{SBP}}$	$0.9971$	$0.9862-1.0081$	$0.594$	$0.9971$	$0.9868-1.0075$	$0.584$
	$\mathrm{Low \, oxygen \, saturation }\;({\text{SpO}}2 <95\mathrm{\%})$	$1.4249$	$0.7476-2.7159$	$0.288$	$1.1910$	$0.6214-2.2826$	$0.600$
$\mathrm{GCS\, score}+{\text{WBC}}$	$\mathrm{GCS \, score}$	$\mathbf{2.5713}$	$\mathbf{1.8692-3.5370}$	$\mathbf{<0.001}$	$\mathbf{3.2651}$	$\mathbf{2.2583-4.7208}$	$\mathbf{<0.001}$
	$\mathrm{WBC \,abnormality}$	$\mathbf{0.3681}$	$\mathbf{0.2077-0.6524}$	$\mathbf{<0.001}$	$\mathbf{0.3882}$	$\mathbf{0.2180-0.6912}$	$\mathbf{0.003}$
$\mathrm{GCS\, score}+{\text{Glucose}}$	$\mathrm{GCS \, score}$	$\mathbf{2.5767}$	$\mathbf{1.9580-3.3909}$	$\mathbf{<0.001}$	$\mathbf{3.0162}$	$\mathbf{2.0178-4.5086}$	$\mathbf{<0.001}$
	$\mathrm{Hyperglycemia }\;({\text{Glucose}}\ge 126\;{\text{mg}}/{\text{dL}})$	$\mathbf{0.2790}$	$\mathbf{0.1237-0.6294}$	$\mathbf{0.001}$	$\mathbf{0.2097}$	$\mathbf{0.0853-0.5155}$	$\mathbf{<0.001}$
$\mathrm{GCS\, score}+{\text{WBC}}+{\text{Glucose}}$	$\mathrm{GCS} \;\mathrm{score}$	$\mathbf{2.4322}$	$\mathbf{1.7716-3.3392}$	$\mathbf{<0.001}$	$\mathbf{2.8445}$	$\mathbf{2.1522-3.7596}$	$\mathbf{<0.001}$
	$\mathrm{WBC \,abnormality}$	$\mathbf{0.4544}$	$\mathbf{0.2489-0.8296}$	$\mathbf{0.003}$	$\mathbf{0.5261}$	$\mathbf{0.2899-0.9549}$	$\mathbf{0.038}$
	$\mathrm{Hyperglycemia }\;({\text{Glucose}}\ge 126\;{\text{mg}}/{\text{dL}})$	$\mathbf{0.3722}$	$\mathbf{0.1478-0.9370}$	$\mathbf{0.018}$	$\mathbf{0.2681}$	$\mathbf{0.0961-0.7480}$	$\mathbf{0.010}$

Values presented in bold indicate statistical significance.

According to the results in Table 4, GCS score is a strong independent dynamic prognostic factor for clinical outcomes. It is calculated that one unit increase in GCS score is associated with 173.79% higher chance of a good outcome when solely jointly modelled with baseline covariates. Presence of WBC abnormality and hyperglycaemia are found to be negatively associated with good outcome at 14 days.

The associations are not significant when these two haematological biomarkers are solely modelled as dynamic covariates but become significant when jointly analysed with GCS score. Moreover, when jointly modelled with haematological biomarkers, the prognostic power of GCS score decreases. These suggest that these two haematological biomarkers can act as confounding variables that affect the associations between GCS score and outcomes. The inclusion of these two haematological biomarkers helps to control for this confounding effect and provide a more accurate estimate of the HR of GCS score.

Vital signs, however, are found not associated with clinical outcomes, with HR values around 1. This may be explained by their dynamic nature, that vital signs can fluctuate and change rapidly in response to various physiological and environmental factors. While abnormal vital signs may indicate physiological instability, they may not necessarily correlate with clinical outcomes. Close monitoring and management of vital signs is important for patients with SAH, but the prognostic value of vital signs for clinical outcomes may be limited compared to neurological scores.

Table 5 gives the prediction performance of different models, where ${T}_{s}$ denotes the starting point of prediction, and $dt$ is the prediction interval. AUC represents the overall discriminative ability of the prognostic model in distinguishing patients with good and poor outcomes. Composition of patients varies with different ${T}_{s}$ and $dt$, which can lead to variations in performance across prediction intervals. Thus we use mean AUC, calculated by averaging the predictive performance over all ${{\text{T}}}_{{\text{s}}}$ and ${\text{dt}}$, to measure the overall prediction performance. Variabilities across starting points of prediction and prediction intervals for each model were also calculated, where low variability indicated the model was robust over time and could consistently provide reliable predictions on clinical outcomes.

**Table 5:** Prediction performance of different joint model settings.

${\text{Covariate}}$	$\mathrm{Baseline\, joint\, model}$						$\mathrm{NIT\, joint\, model}$					
$\mathrm{GCS\, score}$	${\text{dt}}/{\text{Ts}}$	1	2	3	5	7	${\text{dt}}/{\text{Ts}}$	1	2	3	5	7
	1	0.9143	0.7423	0.7016	0.6715	0.7405	1	0.8950	0.7250	0.7039	0.6896	0.7335
	2	0.7441	0.7505	0.6391	0.7561	0.7564	2	0.7432	0.7509	0.6513	0.7521	0.7534
	3	0.7736	0.7294	0.6791	0.7245	0.7544	3	0.7761	0.7383	0.6835	0.7370	0.7685
	5	0.7886	0.7811	0.7046	0.7031	0.7472	5	0.7888	0.7924	0.7255	0.7479	0.7799
	7	0.7865	0.7878	0.7511	0.6968	0.7320	7	0.7831	0.7976	0.7700	0.7603	0.7705
	Marginal	0.8014	0.7582	0.6951	0.7104	0.7461	Marginal	0.7973	0.7609	0.7069	0.7369	0.7611
	$\mathrm{Mean\, AUC}{:} \;0.7422\pm 0.0524$						$\mathrm{Mean\, AUC}{:} \;0.7526\pm 0.0466$					

$\mathrm{GCS\, score}+{\text{Glucose}}$	${\text{dt}}/{\text{Ts}}$	1	2	3	5	7	${\text{dt}}/{\text{Ts}}$	1	2	3	5	7
	1	0.8966	0.7758	0.7285	0.7152	0.7542	1	0.9328	0.7510	0.6984	0.6955	0.7468
	2	0.7795	0.7684	0.6554	0.7588	0.7673	2	0.7563	0.7645	0.6522	0.7521	0.7634
	3	0.7908	0.7546	0.6875	0.7331	0.7632	3	0.7896	0.7581	0.6941	0.7422	0.7669
	5	0.8018	0.8065	0.7205	0.7181	0.7622	5	0.8016	0.8080	0.7294	0.7456	0.7814
	7	0.7975	0.8015	0.7526	0.7168	0.7571	7	0.7954	0.7991	0.7667	0.7497	0.7792
	Marginal	0.8132	0.7814	0.7089	0.7284	0.7608	Marginal	0.8151	0.7761	0.7082	0.7370	0.7675
	$\mathrm{Mean\, AUC}{:} \;0.7585\pm 0.0468$						$\mathrm{Mean\, AUC}{:} \;0.7608\pm 0.0518$					

$\mathrm{GCS \, score}+{\text{WBC}}$	${\text{dt}}/{\text{Ts}}$	1	2	3	5	7	${\text{dt}}/{\text{Ts}}$	1	2	3	5	7
	1	0.8273	0.7714	0.7381	0.7120	0.7476	1	0.8271	0.7597	0.7147	0.7115	0.7436
	2	0.7803	0.7917	0.6647	0.7636	0.7668	2	0.7400	0.7644	0.6589	0.7589	0.7592
	3	0.8000	0.7626	0.7003	0.7392	0.7684	3	0.7938	0.7573	0.6996	0.7416	0.7749
	5	0.8038	0.8095	0.7228	0.7262	0.7675	5	0.8062	0.8137	0.7328	0.7527	0.7903
	7	0.8006	0.8047	0.7589	0.7289	0.7614	7	0.7991	0.8035	0.7731	0.7654	0.7912
	Marginal	0.8024	0.7880	0.7170	0.7340	0.7623	Marginal	0.7932	0.7797	0.7158	0.7460	0.7718
	$\mathrm{Mean\, AUC}{:} \;0.7608\pm 0.0384$						$\mathrm{Mean\, AUC}{:} \;0.7613\pm 0.0390$					

$\mathrm{GCS \, score}+{\text{Glucose}}+{\text{WBC}}$	${\text{dt}}/{\text{Ts}}$	1	2	3	5	7	${\text{dt}}/{\text{Ts}}$	1	2	3	5	7
	1	0.8309	0.7446	0.7176	0.7061	0.7627	1	0.8155	0.7425	0.7520	0.7110	0.7719
	2	0.7815	0.7850	0.6686	0.7626	0.7728	2	0.7469	0.8032	0.6944	0.7767	0.7874
	3	0.8069	0.7684	0.7011	0.7414	0.7700	3	0.8221	0.7879	0.7258	0.7626	0.7892
	5	0.8144	0.8155	0.7340	0.7245	0.7751	5	0.8192	0.8313	0.7636	0.7654	0.7972
	7	0.8061	0.8025	0.7618	0.7196	0.7749	7	0.8138	0.8177	0.7930	0.7715	0.7957
	Marginal	0.8080	0.7832	0.7166	0.7308	0.7711	Marginal	0.8035	0.7965	0.7458	0.7574	0.7883
	$\mathrm{Mean\, AUC}{:} \; 0.7619\pm 0.0407$						$\mathrm{Mean\, AUC}{:} \; 0.7783\pm 0.0356$					

In both baseline and NIT joint models, jointly analysing these three dynamic covariates increases the overall prediction performance compared to solely relying on the GCS score, since WBC and glucose levels can provide additional prognostic information on inflammatory or infectious process, and cardiovascular incidences, to the GCS score, which mainly represents a patient’s level of consciousness and neurological function. Incorporation of multiple dynamic covariates can thus provide a multifaceted view on the evolution of a patient’s clinical status.

Comparing the predictive performance across different prediction intervals, we can find that the worst predictive performance for all model settings is predicting from day 3, especially when predicting the outcome in the next two days. This can be explained by both the nature of spontaneous SAH and the dataset. Firstly, regarding the nature of spontaneous SAH, complications, e.g., vasospasm. are highly probable within this time period^16^. Thus lack of prognostic information about the time to complications during this critical period hinders our model's ability to capture essential prognostic information, leading to reduced predictive performance. Secondly, calculations of AUCs are based on the comparison between model’s predictions and actual outcomes within the prediction interval and can be impacted by few outliers or atypical cases. Therefore, we measure and compare the overall performance acorss different model settings by averaging AUCs across multiple prediction periods.

**Figure 2:**
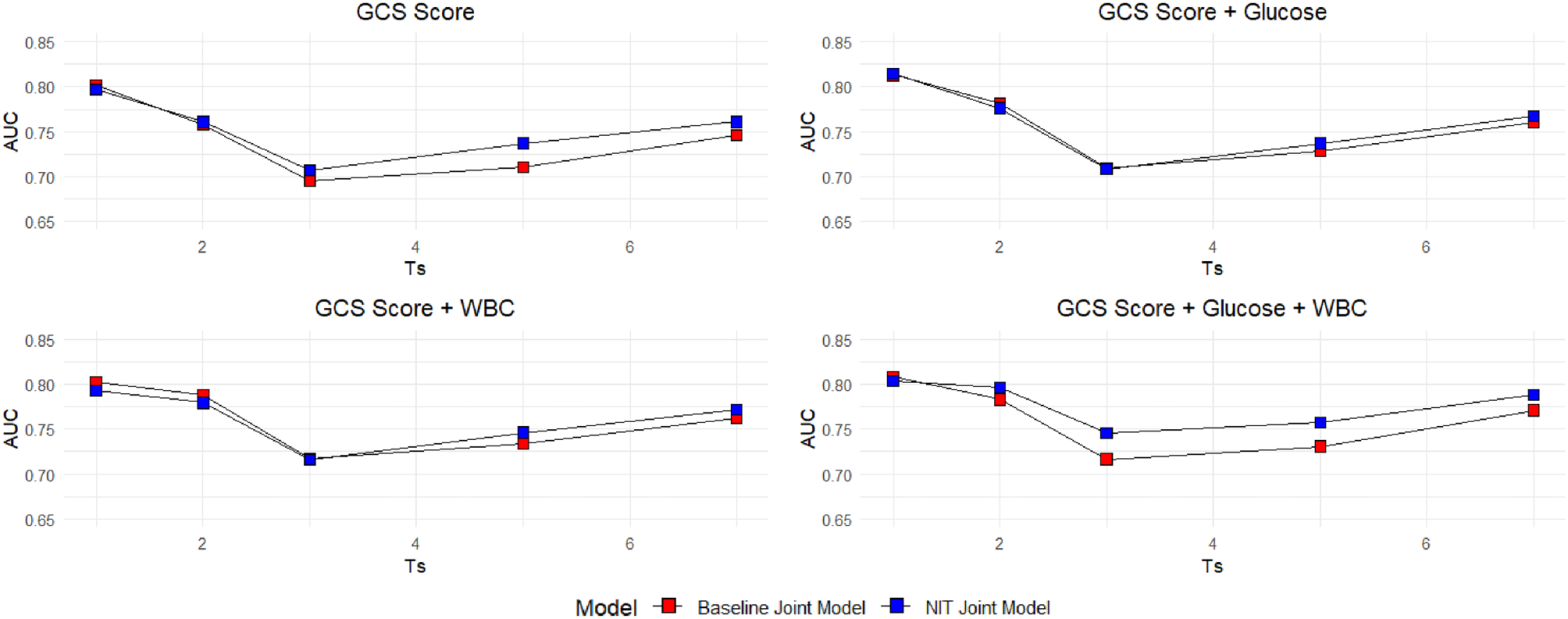
Comparisons in prediction accuracy between baseline and NIT joint models.

Figure 2 compares the prediction accuracy with time between two modelling approaches and combinations of dynamic covariates, measured by marginal AUC by the time of prediction. It can be found that, with all combinations of dynamic covariates, the prediction performance of proposed NIT joint model is better than the corresponding model developed by baseline joint modelling methods. This figure also shows that the prediction performance of proposed NIT joint model with GCS score, glucose and WBC as dynamic covariates is good and consistent with different prediction time, where the AUC is at least around 0.75 for all starting points of prediction. Moreover, prognostic NIT joint model can provide good prediction performance in both acute phase (${T}_{s}+dt\le 3$, Mean AUC: 0.7683) and sub-acute phase ($3<{T}_{s}+dt\le 14$, Mean AUC: 0.7797) of spontaneous SAH.

According to the predictive performance of different dynamic prognostic models, there are three main findings of this study. Firstly, repeated measured neurological status, measured by GCS score, is a strong dynamic predictor for clinical outcomes, adjusting for baseline clinical conditions. Incorporation of haematological biomarkers, i.e., WBC and glucose, can provide additive prognostic information and improve the prediction accuracy of prognostic models.

Secondly, intermediate events, i.e., neurological interventions in this study, provide prognostic information on the disease progression. Incorporating intermediate events as transitions between states of prognostication can add granularity to a prognostic model by dividing the overall prognosis process into multiple prognostic states, which allows for a more detailed analysis into disease progression. Compared with baseline joint models, NIT joint models have better predictive accuracy when including the same baseline and dynamic covariates.

Moreover, NIT joint models contribute to personalised prognosis. Modelling the time point of neurological intervention as individualised transition point of prognostic states adds individual-specific characteristics to the prognostic model so that a prognostic model can take individualised milestones in disease progression into account for personalised outcome predictions.

Finally, compared to baseline joint models, NIT joint models perform better in relative long-term outcome prediction. The average improvement of prediction accuracy from baseline to NIT joint model across four covariate combinations is 0.0212 for prediction in the sub-acute phase, while the overall improvement is 0.0074. This merit of NIT joint model may result from that it explains the change of disease progression, which has higher impact on the values of dynamic covariates in the sub-acute phase of prognostication.

The multivariate NIT joint model, incorporating GCS score, WBC and glucose as dynamic prognostic covariates is the optimal model in this study, increasing the predictive accuracy of outcome from 0.7422, in baseline joint model only considering the neurological grades of patients, to 0.7783. It can be used as an accurate and comprehensive clinical tool for dynamic personalised prognosis in patients suffering from spontaneous SAH, potentially benefiting disease progression monitoring, optimising treatment plans for better clinical outcomes.

## Discussion

This study has proposed a multivariate NIT joint model for prognosis of spontaneous SAH. Compared to widely used clinical tools such as Hunt and Hess grade, which are highly dependent on patient’s neurological status, the prognostic model explores the prognostic values of medical conditions, haematological biomarkers, and events of neurological interventions. It is thus suitable for modelling the multi-factorial mechanism of SAH prognosis. Moreover, prognostic information from medical conditions and haematological biomarkers are individual-specific, together with individualised disease progression modelled by individualised prognostic state transitions, making proposed NIT models in this study a good clinical tool for personalised prognosis.

The optimal NIT joint model is composed of five baseline covariates, i.e., cerebral oedema, cerebral infarction, respiratory failure, hydrocephalus and vasospasm, three dynamic covariates, i.e., GCS score, WBC and glucose, and a state transition indicator, i.e., intermediate event of neurological intervention. This model can provide accurate outcome predictions across all starting points of prediction and prediction intervals, with the overall AUC 0.7783, respectively 0.7683 and 0.7797 in the acute and sub-acute phases of spontaneous SAH.

In analysis of the predictive power of neurological status, haematological biomarkers, and vital signs, GCS score was found to be the most valuable covariate in prognostication. This finding was consistent with the fact that neurological scales are widely used clinical tools for prognosis of SAH, which estimated patients’ clinical outcomes based on results in neurological assessments. WBC and glucose are not independent prognostic factors but can provide additive prognostic information to neurological status and improve the model prediction accuracy.

Although the prognostic values of vital signs were found to be limited in this study, their variability, e.g., systolic blood pressure variability, which may indicate impaired blood pressure regulation and cardiovascular instability, is drawing research attention in prognosis studies^17^. With more frequent measurements of vital signs, their variability and the changes of variability during disease progression can be obtained, and can be potentially included as influential dynamic covariate for the prognostication of SAH.

This study provides a novel approach to incorporate different types of covariates and events into prognostication of spontaneous SAH, on the basis of joint modelling framework. This approach is also suitable and can be extended to model the prognosis of other cerebrovascular diseases due to its ability to incorporate different types of prognostic information. Incorporated prognostic information is from demographics, clinical conditions, neurological status, haematological biomarkers, vital signs, radiological findings, and some intermediate events that can have a direct impact on disease progression such as complications and neurological surgeries. Moreover, the Bayesian approach adopted for parameter estimation allows for incorporating expert knowledge into prior distributions, which facilitates its clinical use and improves model’s interpretability for clinicians.

The findings of this study also contribute to the development of personalised prognosis, which is a tendency in critical care management. Personalised prognosis considers individual characteristics and can guide tailored treatment decisions, individualised follow-up and surveillance strategies, and resource allocation. Implementation of personalised prognosis in clinical practice by integration with EHR systems can help improve patient outcomes, enhance the collaboration between patients and caregivers, and support more patient-centred healthcare. This study is the first research taking all three types of individual-specific covariates and events, i.e., baseline clinical conditions, dynamic prognostic factors and clinical intermediate events, into prognostic modelling in studies of neurovascular diseases. Findings and approaches proposed in this study can thus potentially contribute to the development of personalised prognosis for neurovascular diseases (supplementary information file 1).

There are three main future directions of this study. Firstly, neurological intervention is not the only intermediate event that can provide additive prognostic information to baseline and dynamic covariates. Occurrences of major complications during critical care, e.g., re-bleeding, and delayed cerebral ischaemia (DCI), are also informative indicators for changes in disease progression, which often indicate worsening neurological conditions. Incorporating multiple intermediate outcomes and development of a multi-state prognostic model can potentially provide more accurate and comprehensive prognostication. Secondly, we have incorporated daily average values of dynamic covariates into prognostic modelling. Thus, our prognostic model may not fully capture within-day changes in patients’ conditions, and the precision of predictions may be impacted, which can be improved with increased frequencies in measuring dynamic covariates. Finally, application of deep learning strategies in the prognosis of spontaneous SAH can potentially help capture informative features for prognosis research, thus improving model’s flexibility and adaptability, which can be potentially integrated with our proposed modelling framework for higher predictive performance^18^.

### Supplementary Information

Supplementary Information.

## Data Availability

All data generated or analysed during this study are included in this published article and its supplementary information file. MIMIC-IV dataset is publicly available on https://physionet.org/content/mimiciv/.

## References

[1] Ingall T, Asplund K, Mähönen M, Bonita R (2000). A multinational comparison of subarachnoid hemorrhage epidemiology in the WHO MONICA stroke study. Stroke.

[2] Hunt WE, Hess RM (1968). Surgical risk as related to time of intervention in the repair of intracranial aneurysms. J. Neurosurg..

[3] Teasdale GM, Drake CG, Hunt W, Kassell N, Sano K, Pertuiset B, De Villiers JC (1988). A universal subarachnoid hemorrhage scale: Report of a committee of the World Federation of Neurosurgical Societies. J. Neurol. Neurosurg. Psychiatry.

[4] Fisher CM, Kistler JP, Davis JM (1980). Relation of cerebral vasospasm to subarachnoid hemorrhage visualized by computerized tomographic scanning. Neurosurgery.

[5] Frontera JA, Claassen J, Schmidt JM, Wartenberg KE, Temes R, Connolly ES, Macdonald RL, Mayer SA (2006). Prediction of symptomatic vasospasmafter subarachnoid hemorrhage: the modified fisher scale. Neurosurgery.

[6] Witsch J, Frey HP, Patel S, Park S, Lahiri S, Schmidt JM, Agarwal S, Falo MC, Velazquez A, Jaja B, Macdonald RL (2016). Prognostication of long-term outcomes after subarachnoid hemorrhage: The FRESH score. Ann. Neurol..

[7] Johnson AE, Bulgarelli L, Shen L, Gayles A, Shammout A, Horng S, Pollard TJ, Hao S, Moody B, Gow B, Lehman LWH (2023). MIMIC-IV, a freely accessible electronic health record dataset. Scientific Data.

[8] Rosen DS, Macdonald RL (2005). Subarachnoid hemorrhage grading scales: A systematic review. Neurocritical Care.

[9] Lampmann T, Hadjiathanasiou A, Asoglu H, Wach J, Kern T, Vatter H, Güresir E (2022). Early serum creatinine levels after aneurysmal subarachnoid hemorrhage predict functional neurological outcome after 6 months. J. Clin. Med..

[10] Ma, X., Lan, F. & Zhang, Y. Associations between C-reactive protein and white blood cell count, occurrence of delayed cerebral ischemia and poor outcome following aneurysmal subarachnoid hemorrhage: A systematic review and meta-analysis. *Acta Neurologica Belgica*, pp. 1–14 (2021) 10.1007/s13760-020-01496-y PMC7796813 33423218

[11] Schmutzhard E, Rabinstein AA, Participants in the International multi-disciplinary Consensus Conference on the Critical care Management of Subarachnoid Hemorrhage (2011). Spontaneous subarachnoid hemorrhage and glucose management. Neurocritical Care.

[12] Rosengart AJ, Schultheiss KE, Tolentino J, Macdonald RL (2007). Prognostic factors for outcome in patients with aneurysmal subarachnoid hemorrhage. Stroke.

[13] Ryttlefors M, Howells T, Nilsson P, Ronne-Engström E, Enblad P (2007). Secondary insults in subarachnoid hemorrhage: Occurrence and impact on outcome and clinical deterioration. Neurosurgery.

[14] Singh R, Mukhopadhyay K (2011). Survival analysis in clinical trials: Basics and must know areas. Perspect. Clin. Res..

[15] Tsiatis AA, Degruttola V, Wulfsohn MS (1995). Modeling the relationship of survival to longitudinal data measured with error. Applications to survival and CD4 counts in patients with AIDS. J. Am. Stat. Assoc..

[16] Psychogios K, Tsivgoulis G, FESO F (2019). Subarachnoid hemorrhage, vasospasm, and delayed cerebral ischemia. Pract. Neurol..

[17] Lin, Q. S., Lin, Y. X., Lin, Z. Y., Yu, L. H., Dai, L. S. & Kang, D. Z. Systolic blood pressure variability is a novel risk factor for rebleeding in acute subarachnoid hemorrhage: a case–control study. *Medicine*, 95(11) (2016) 10.1097/MD.0000000000003028 PMC4839899 26986118

[18] Lin J, Luo S (2022). Deep learning for the dynamic prediction of multivariate longitudinal and survival data. Stat. Med..

